# Initial Experience Performing Mechanical Thrombectomy With the CatchView Mini Device for Distal M2 Segment Middle Cerebral Artery Occlusions

**DOI:** 10.3389/fneur.2021.724811

**Published:** 2021-09-14

**Authors:** Pedro Vega, Eduardo Murias, Jose María Jimenez, Juan Chaviano, Lorena Benavente, Montserrat Gonzalez-Delgado, Faustino García-Arias, José Manuel Pumar

**Affiliations:** ^1^Department of Radiology, Central University Hospital of Asturias, Oviedo, Spain; ^2^Cátedra Institucional de Neurorradiología Intervencionista, Universidade de Santiago de Compostela, Santiago de Compostela, Spain; ^3^University of Oviedo, Oviedo, Spain; ^4^Department of Neurology, Central University Hospital of Asturias, Oviedo, Spain

**Keywords:** stroke, thrombectomy, stent, hemorrhage, endovascular treatment

## Abstract

**Background:** Mechanical thrombectomy (MT) has become the standard of care for acute ischemic stroke due to large vessel occlusion; however, its safety and efficacy in patients with distal strokes remains unclear. In this study, we investigated the safety and efficacy of MT for distal middle cerebral artery (MCA) occlusions using the CatchView Mini (CVM; Balt, Montmorency, France).

**Methods:** This was a prospective single-center analysis of patients with a single MCA-M2 occlusion treated with the CVM device. Consecutive patients were prospectively enrolled from October 2018 to March 2020. Efficacy outcomes included successful recanalization [modified Thrombolysis in Cerebral Infarction (mTICI) 2b/3], procedure times, and number of device passes. Clinical outcomes included National Institutes of Health Stroke Scale Score (NIHSS) at discharge, 90-day functional independence (modified Rankin Scale 0–2) and safety outcomes included hemorrhagic complications, and 90-day mortality.

**Results:** A total of 45 patients (mean age: 74.0 ± 12.6; 53.3% [24/45] female) were included in the study. Upon admission, 33.3% (15/45) of patients were mRS 3–5; and mean NIHSS was 13.2 ± 4.2 Mean time from symptom onset to final angiography was 250.0 ± 83.4 min with a mean intervention duration of 34.0 ± 12.6 min. The mean number of device passes was 1.8 ± 1.5 final mTICI 2b/3 was achieved in 91.1% (41/45) of patients. Eight hemorrhagic complications (17.8%, 8/34) occurred, none of which were symptomatic. At 90-days, 57.8% (26/45) patients were functionally independent and the rate of mortality was 15.6% (7/45).

**Conclusions:** The present analysis demonstrates a low risk profile and high recanalization success for patients with distal M2 occlusions treated with the CVM device.

## Introduction

Major randomized clinical trials have established the superiority of mechanical thrombectomy (MT) to medical management in patients with acute ischemic stroke due to large vessel occlusion of the anterior circulation (1–7). Newer generation MT devices have allowed operators to navigate more distally and with higher technical precision, expanding the potential therapeutic applications of MT. Yet, current evidence supporting the safety and efficacy of MT for distal occlusion is limited given the exclusion of these patients in major clinical trials. A recent meta-analysis of patients with occlusion of the M2 segment of the middle cerebral artery (MCA) demonstrated a benefit of MT over best medical care for 90-day modified Rankin Scale (mRS) score, but other apparent benefits were not statistically significant (8). M2 occlusions have also been associated with a higher rate of revascularization after treatment with intravenous tissue plasminogen activator (rTPA) compared to more proximal occlusions (9). Accordingly, available international guidelines do not recommend MT for distal occlusions based on the current level of scientific evidence (10).

The CatchView Mini (CVM; Balt, Montmorency, France) is a new version of the low-profile, laser-cut nitinol Catch+ Mini (Balt, Montmorency, France) stent retriever (SR) that boasts improved visibility and device length, as well as an overlapping structure that yields good adaptability to arterial conformation with less rectification during the clot extraction. The aim of this research was to examine the safety and efficacy of MT for occlusions of the M2 or more distal segments of the MCA. We investigated clinical, radiologic, and safety outcomes after MT with the CVM device.

## Methods

### Study Design and Patient Selection

The study was a prospective single-center analysis of clinical, radiologic, and safety outcomes after MT as primary treatment for occlusions of the M2 or more distal segments using the CVM. The study enrolled consecutive patients from October 2018 to March 2020. The M2 branch was defined in accordance with anatomic boundaries, in which the M2 segment begins at the genu of the MCA and extends laterally toward the Sylvian fissure. Patients with proximal occlusions were excluded. The study was approved by the institutional ethics board and all patients provided written informed consent. In that cases in which the patient couldn't sign the informed consent, a Legally Authorized Representative signed it.

### Baseline Characteristics

Patient baseline evaluation assessed age, sex, anticoagulant treatment, cardiovascular risk factors, pre-stroke mRS, initial National Institutes of Health Stroke Scale (NIHSS), and Alberta Stroke Program Early Computed Tomography Score (ASPECTS), the presence of CT perfusion mismatch, and treatment with intravenous recombinant tissue plasminogen activator (rTPA) before thrombectomy.

### Endovascular Procedure

**Figure 1:**
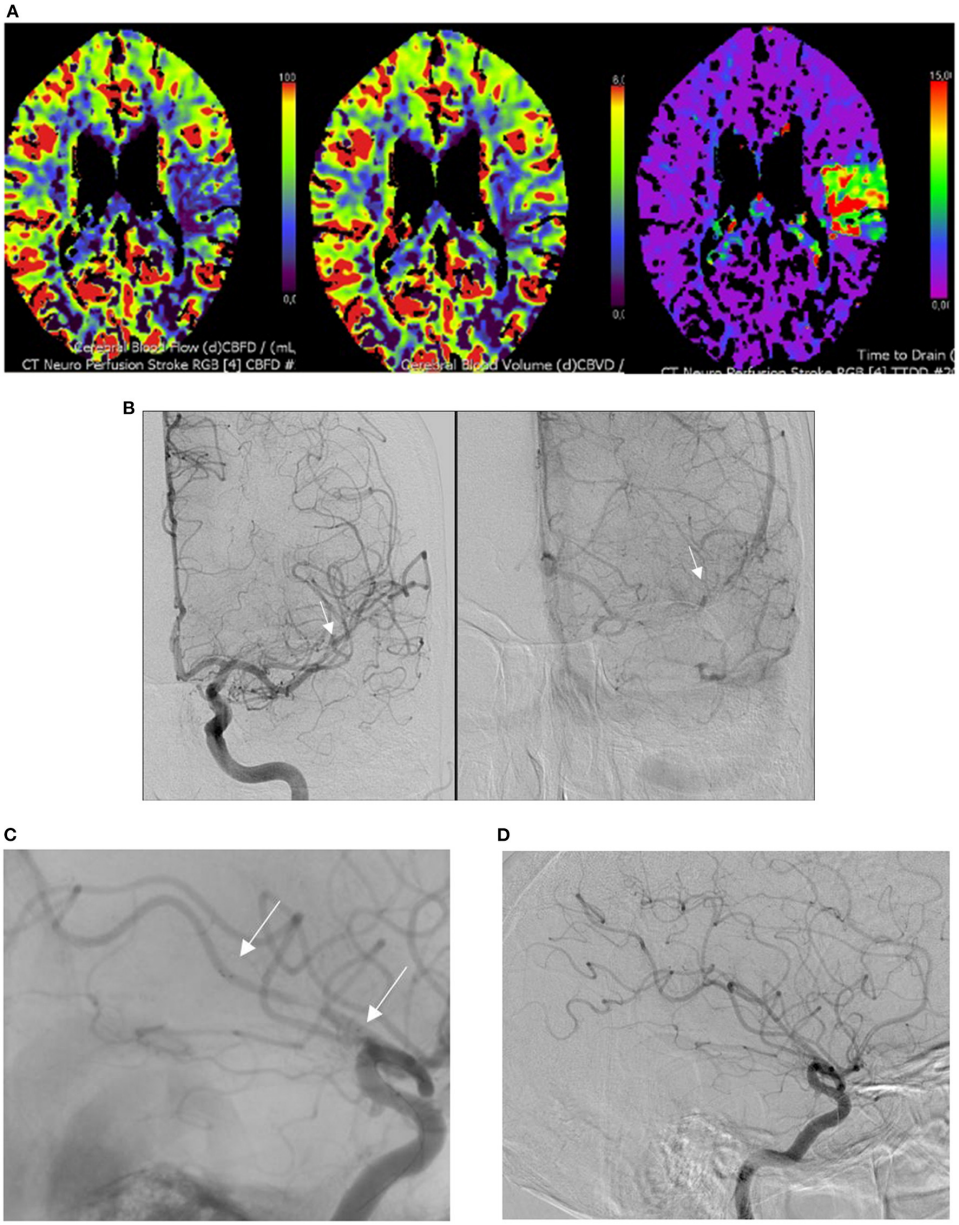
M2 occlusion treated with the CatchView Mini device. **(A)** Perfusion computed tomography demonstrating a decrease in blood flow with normal volume and an increase in mean transit time in the territory of the left M2 segment of the middle cerebral artery. **(B)** Visualization of the M2 occlusion on angiography (anteroposterior view, white arrows). **(C)** Visualization of the distal and proximal ends of the opened CatchView Mini stentriever device inside of the clot on angiography (white arrows). **(D)** Final angiography post-procedure demonstrating complete recanalization of the occluded vessel.

All procedures were performed using a biplane angiography machine. An 8F balloon catheter was positioned in the internal carotid artery. Navigation to the target vessel was accomplished using a 0.014-inch Traxcess guidewire (Microvention, Aliso Viejo, CA) in a Headway duo microcatheter (Microvention, Aliso Viejo, CA). After passage through the clot, intraarterial contrast medium was injected to verify the position of the microcatheter distal to the clot. In cases of primary occlusion of a small vessel feeding eloquent brain areas, the CVM (20 mm length) was used as a front-line device. No adjunctive treatments were used. The ST was deployed by withdrawal of the microcatheter and contrast was injected to evaluate flow after placement of the device. Two to three minutes thereafter, the proximal balloon guiding catheter was inflated to arrest blood flow and the open ST was removed with aspiration (Figure 1).

### Procedural and Clinical Outcomes

Procedural variables included the modified Thrombolysis in Cerebral Infarction (mTICI) score as a measure of target artery reperfusion (11). All angiograms were adjudicated by an independent physician and successful recanalization was defined as a mTICI score of 2b/3 (complete or near-complete recanalization). We also analyzed the mean treatment time (stroke symptom onset to final angiography), mean intervention time (arterial puncture to final angiography), and mean number of device passes. Vasospasm after retraction of the device was also noted and defined as >50% stenosis on follow-up angiography. Other analyzed variables included vessel perforation, contrast extravasation, and embolism in a new vascular territory.

Clinical outcomes included mean NIHSS score at discharge and 90-day functional independence (mRS score of 0–2 at 90 days). Clinical safety outcomes included 90-day mortality, intracranial hemorrhage, and symptomatic intracerebral hemorrhage (SICH) defined as any type of hemorrhage with an increase of ≥4 in NIHSS score. Hemorrhagic complications were identified in accordance with the European Cooperative Acute Stroke Study III criteria (12).

### Statistical Analysis

Descriptive statistics were generated using SPSS 17.0 software. Categorical variables are presented as absolute values and percentages and continuous variables are presented as means and standard deviations.

## Results

Patient baseline characteristics are described in Table 1 and study outcomes are presented in Table 2.

**Table 1:** Patient baseline characteristics.

**Characteristic**	**Overall (** ***N*** **=45)**
Age	74.0 ± 12.6
Female	24 (53.3%)
**Cardiovascular comorbidities**	
Hypertension	33 (73.3%)
Atrial fibrillation	31 (68.9%)
**mRS**	
0–2	30 (66.7%)
3–5	15 (33.3%)
**NIHSS**	
0	0 (0%)
1–4	0 (0%)
5–15	33 (73.3%)
16–20	12 (26.6%)
Mean (SD)	13.2 ± 4.2
ASPECTS	9.0 ± 1.0
CT perfusion mismatch (*N* = 42)	21 (50.0%)
IV rTPA	14 (31.1%)
**Occlusion side**	
Right	15 (33.3%)
Left	30 (66.7%)
Midline	0 (0%)

*Data are N (%), mean ± SD, or median (minimum-maximum). ASPECTS, Alberta Stroke Program Early CT Score; IV rTPA, intravenous recombinant tissue plasminogen activator; mRS, modified Rankin Scale; NIHSS, National Institute of Health Stroke Scale*.

**Table 2:** Procedural and clinical characteristics.

**Characteristic**	**Overall (** ***N*** **= 45)**
Treatment time^a^ (min)	250.0 ± 83.4
Intervention time^b^ (min)	34.0 ± 12.6
Device passes	1.8 ± 1.5
1	28 (62.2%)
2	9 (20%)
3	4 (8.8%)
Final TICI 2b/3	41 (91.1%)
**Hemorrhagic complications**	
Subarachnoid hemorrhage	3 (6.7%)
Type-1 intraparenchymal hemorrhage	5 (11.1%)
**Procedural complications**	
Vessel perforation	0 (0.0%)
Contrast extravasation	0 (0.0%)
Embolism in new vascular territory	0 (0.0%)
NIHSS at discharge	5.0 ± 6.6
0	14 (31.1%)
1–4	13 (28.8%)
5–15	12 (26.6%)
16–20	1 (2.2%)
90-day mortality	7 (15.6%)
90-day mRS 0–2	26 (57.8%)

*Data are N (%), mean ± SD, or median (minimum–maximum)*.

a *Treatment time is defined as the time from stroke symptom onset to final angiography*.

b *Intervention time is defined as the time from arterial puncture to final angiography*.

*mRS, modified Rankin Scale; NIHSS, National Institute of Health Stroke Scale; TICI, Thrombolysis in Cerebral Infarction*.

### Baseline Characteristics

The study included 45 patients who presented with a single M2 occlusion on initial angiography. The population was 53.3% female (24/45) with a mean age of 74.0 ± 12.6 years; 46.7% (21/45) of patients were older than 80 years of age. Most occlusions (66.7% [30/45]) were located on the left side. The most common cardiovascular risk factor was hypertension, present in 73.3% (33/45) of patients, followed by atrial fibrillation in 68.9% (31/45) of patients. At admission, 33.3% (15/45) of patients had a mRS score of 3–5; mean NIHSS score was 13.2 ± 4.2; and mean ASPECTS score was 9.0 ± 1.0. Computerized tomography (CT) perfusion was performed in 93.3% (42/45) of patients; of these, 50.0% (21/42) exhibited perfusion mismatch. CT perfusion could not be performed in 3 cases due to patient agitation. Fifteen patients (33.3% [15/45]) were receiving anticoagulant treatment and 14 (31.1% [14/45]) were treated with at least a bolus of intravenous rtPA before the procedure. Fourteen patients (31.1% [14/45]) were treated under general anesthesia.

### Procedural Characteristics and Efficacy Outcomes

The mean time from symptom onset to final angiography was 250 min with a mean intervention time of 34 min. The mean number of device passes was 1.8. No patients necessitated rescue therapy. Target artery reperfusion was assessed in all 45 patients and yielded a recanalization rate (TICI 2b/3) of 91.1% (41/45).

### Safety and Clinical Outcomes

There were eight (17.8% [8/45]) hemorrhagic complications (3 subarachnoid hemorrhages and 5 type-1 intraparenchymal hemorrhages), but none were symptomatic. There were no instances of vessel perforation, contrast extravasation, or embolism in a new vascular territory. The mean NIHSS score at discharge was 5 and the 90-day mortality rate was 15.6% (7/45). Twenty-six patients (57.8% [26/45]) were functionally independent (mRS 0–2) at 90 days.

## Discussion

In the present study, a majority of patients treated with the CVM device for an M2 occlusion achieved successful reperfusion (TICI 2b/3). MT was also associated with a low risk of complications and a 90-day mortality rate consistent with those reported in previous clinical trials. Taken together, our findings support the safety and efficacy of MT with the CVM for distal occlusion of the MCA.

The rate of successful recanalization in this study was notably higher than that reported after intravenous alteplase in a study of patients with M2 segment MCA occlusions (37.1%) (13) and in the HERMES meta-analysis (59.2%) (8). A high rate of reperfusion in the present study may be in part attributable to our treatment protocol, which implements a proximal balloon guiding catheter together with a new-generation, long (20 mm), low-profile ST that offers better flexibility, adaptability, and navigability for treating small and tortuous vessel occlusions than STs typically used to treat proximal occlusions. Previous research suggests that ST length influences recanalization success (14). Moreover, the CVM is fully compatible with the low-profile Headway Duo microcatheter (1.3F distally, 0.012″ internal lumen), which has been previously demonstrated to reduce the likelihood of clot migration during MT (15).

We similarly observed good clinical outcomes in our study, with more than half patients exhibiting functional independence (mRS 0–2) at 90 days post-procedure. In the HERMES meta-analysis, the direction of clinical benefit generally favored endovascular treatment over medical treatment (mRS 0–2 at 90 days 58.2 vs. 39.7%, respectively), but the results were not statistically significant for all outcomes (8). A larger benefit observed in our study may be due to differences between studies in the included patient population and the use of different treatment protocols, as the HERMES analysis included a broad variety of intra-arterial therapies. Moreover, the meta-analysis results may have been underpowered given the small, pooled sample size of patients with M2 occlusion. Finally, divergence between our results and those of the meta-analysis may have been related to differing definitions as to what constitutes M1 occlusion vs. M2 occlusion from an anatomical perspective (9).

MT with the CVM in our study produced a low rate of complications. There were no observed cases of SICH and relatively few cases of subarachnoid hemorrhage and type-1 intraparenchymal hemorrhage. Additionally, there were no cases of vessel perforation or extravasation. These results are consistent with those reported by the HERMES meta-analysis, which did not identify any SICH or major procedural complications in the endovascular treatment group vs. a cumulative rate of 7.9% in the control arm (8). In contrast, a meta-analysis by Saber et al. (16) reported a 10% rate of SICH after MT for distal occlusion. A higher rate of SICH in the aforementioned study may reflect the risk associated with the use of devices designed for proximal occlusion to treat smaller or more fragile vessels. Furthermore, Baharvahdat et al. (17) examined the rates of post-thrombectomy subarachnoid hemorrhage based on occlusion location and described a higher rate in the M2 group compared to the M1 group (25 vs. 12%; *P* = 0.010); however, this difference was not associated with significant long-term clinical consequences. In our series, we detected a lower rate of subarachnoid hemorrhage with no observed worsening of the patients' clinical condition.

MT in our study was not associated with vessel vasospasm in any patient. Vessel vasospasm was detected in a significant number of patients (22.5%) who underwent treatment with a ST in the SWIFT trial (18) and in more than half of the patients in the Baby Trevo series (19) without clinical sequelae, which may indicate that this is not a hazardous event. Nonetheless, intra-arterial vasodilator infusion either prophylactically or to treat angiographic vasospasm produced good results in these studies. Finally, it is noteworthy that the 90-day mortality rate observed in our study is comparable to that reported in previous trials [11.9% (8) and 15.8% (16)].

The present study has several limitations. First, we included a relatively small sample size. Given a paucity of corroborating evidence for the safety and efficacy of our treatment protocol, additional data is necessary to support MT as a therapeutic option for distal MCA occlusion in international guidelines. Second, the present research was a prospective case series and, therefore, lacked randomization and a control arm for comparison. It is important to acknowledge that a careful risk benefit assessment is important, especially in a context of distal occlusion. Future efforts should include a randomized controlled trial of MT vs. medical therapy for M2 occlusion; however, this may not be feasible due to the lack of clinical equipoise, as MT is now routinely performed for M2 occlusion in contrast with guideline recommendations. Finally, it is important to underscore that our study findings were likely bolstered by use of the CVM device, which offers key advantages for the treatment of distal occlusions relative to STs employed for the treatment of proximal occlusions.

## Conclusions

Our analysis demonstrates that, in a context of distal M2 occlusion, MT with the CVM device is associated with a low risk profile and a high rate of recanalization success.

## Data Availability Statement

The raw data supporting the conclusions of this article will be made available by the authors, without undue reservation.

## Ethics Statement

The studies involving human participants were reviewed and approved by Hospital Universitario Central de Asturias: Ethics Committee. The patients/participants provided their written informed consent to participate in this study.

## Author Contributions

PV: responsible for the integrity of the study, study design, data collection, analysis and interpretation of data, statistical treatment, literature research, text writing, critical revision, and approval of the final version. EM: critical revision, approval of the final version, and responsible for the integrity of the study. JJ and JC: text writing, data collection, study design, and approval of the final version. MG-D, LB, FG-A, and JP: critical revision and approval of the final version. All authors contributed to the article and approved the submitted version.

## Conflict of Interest

The authors declare that the research was conducted in the absence of any commercial or financial relationships that could be construed as a potential conflict of interest.

## Publisher's Note

All claims expressed in this article are solely those of the authors and do not necessarily represent those of their affiliated organizations, or those of the publisher, the editors and the reviewers. Any product that may be evaluated in this article, or claim that may be made by its manufacturer, is not guaranteed or endorsed by the publisher.
